# Circulating immune cell composition and activation status associate with brain white matter microstructure in bipolar depression

**DOI:** 10.1192/j.eurpsy.2023.151

**Published:** 2023-07-19

**Authors:** F. Benedetti

**Affiliations:** ^1^ University Vita-Salute San Raffaele; ^2^Division of Neuroscience, Scientific Institute Ospedale San Raffaele, Milano, Italy

## Abstract

**Abstract:**

Bipolar disorder (BD) has been consistently associated with alterations in the immune system. Evidence suggests a condition of systemic low-grade inflammation due to decreased adaptive, increased innate immunity, with higher levels of circulating cytokines, higher macrophage/monocyte inflammatory activation patterns, and higher neutrophils to lymphocyte counts; and with a dynamic pattern of premature immunosenescence and partial T cell defect starting early in adolescence, involving a reduction of naïve T cells and an expansion of memory and senescent T cells. Quantitative analysis of circulating inflammatory markers suggested persistent low-grade inflammation.

A growing literature suggests that the immune system plays a core role in maintaining brain homeostasis, with both adaptive and innate immune support, ensured by cell trafficking across the blood brain barrier, being essential for brain maintenance and repair in healthy conditions, and disrupted in brain disorders including BD. Measured in peripheral blood, these markers of altered immuno-inflammatory setpoints parallel activation of microglia and disruption of white matter (WM) integrity in the brain.

Studies in the field are in its infancy, but findings by our group showed that: circulating Th17 cells correlated with higher FA, while regulatory FOXP3^+^ cells correlated with higher RD and MD, and with lower fMRI neural responses in the right dorsolateral prefrontal cortex; higher circulating cytokine-producing NK cells were fostered by ongoing lithium treatment and directly correlated with better FA, and inversely with RD and MD, also partially mediating the known benefits from lithium on WM; and activation status and expression of killer proteins by cytotoxic CD8^+^ T cells negatively associated with WM microstructure, thus suggesting that CD8^+^ T cells can leave the blood stream to migrate into the brain and induce an immune-related WM damage in BD.

Implications of these findings for neuroprogression, clinical outcomes, and new treatment strategies of the disorder will be discussed.

**Disclosure of Interest:**

None Declared

